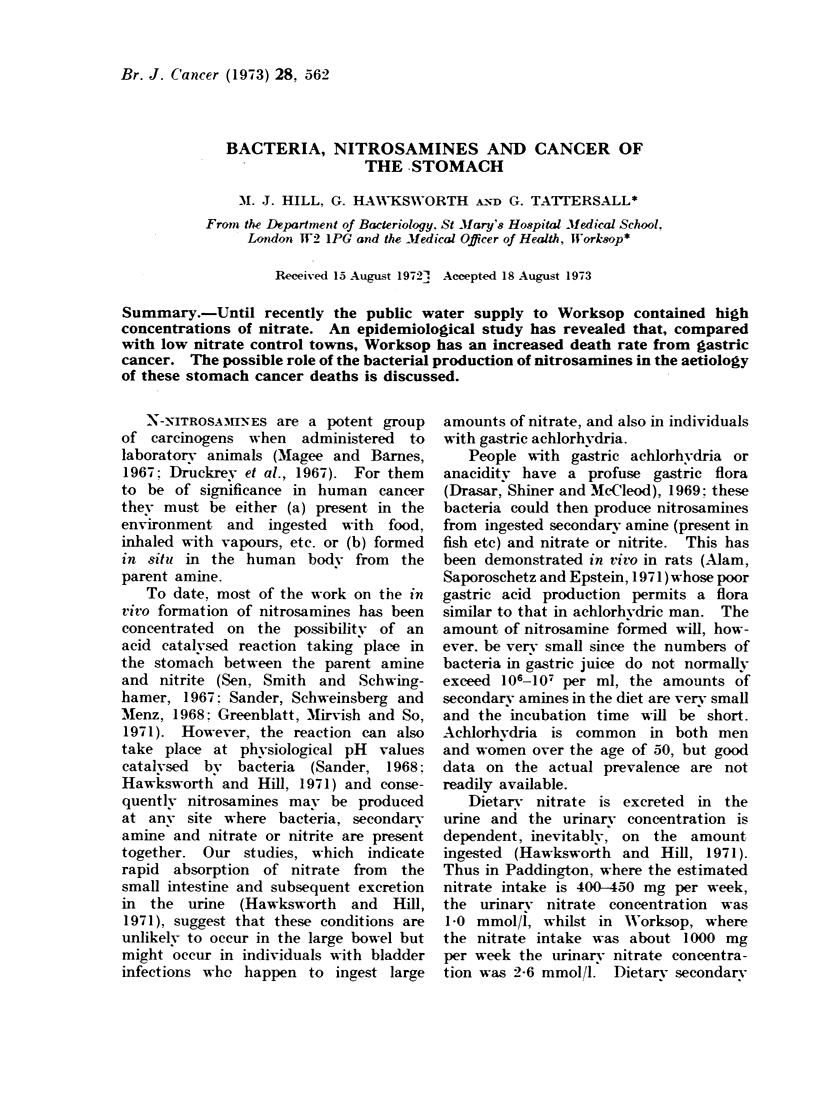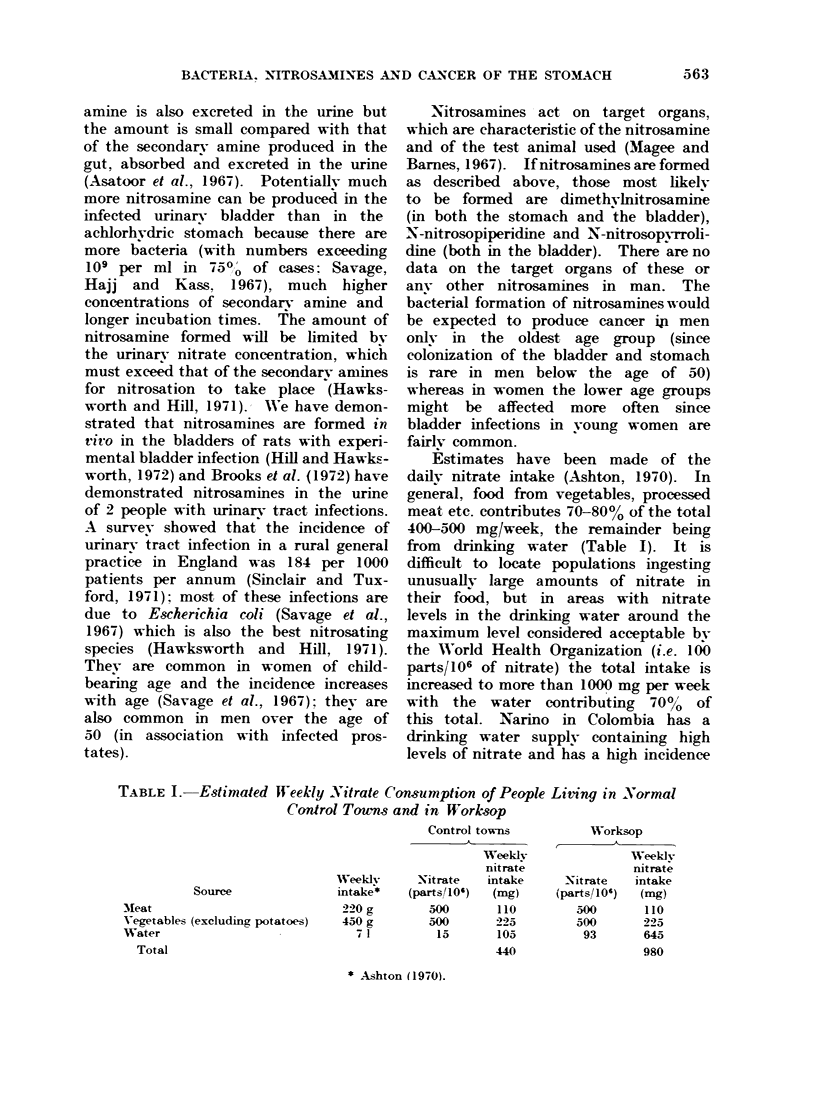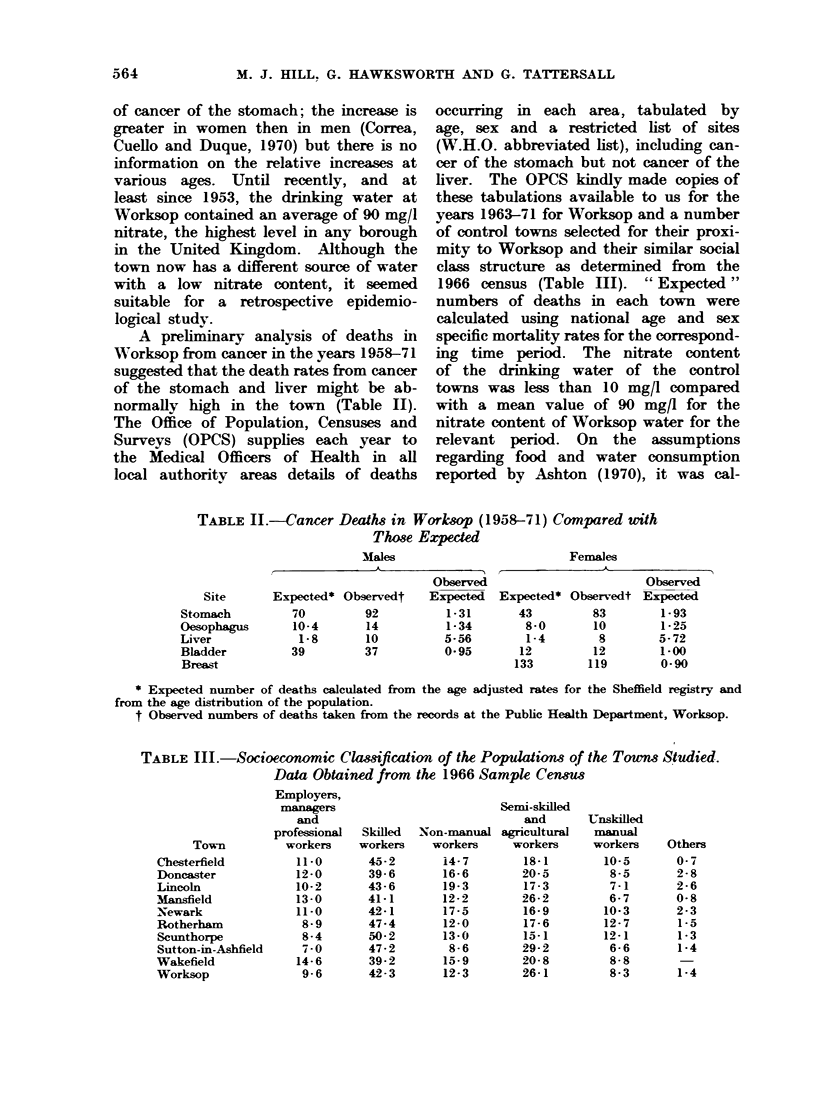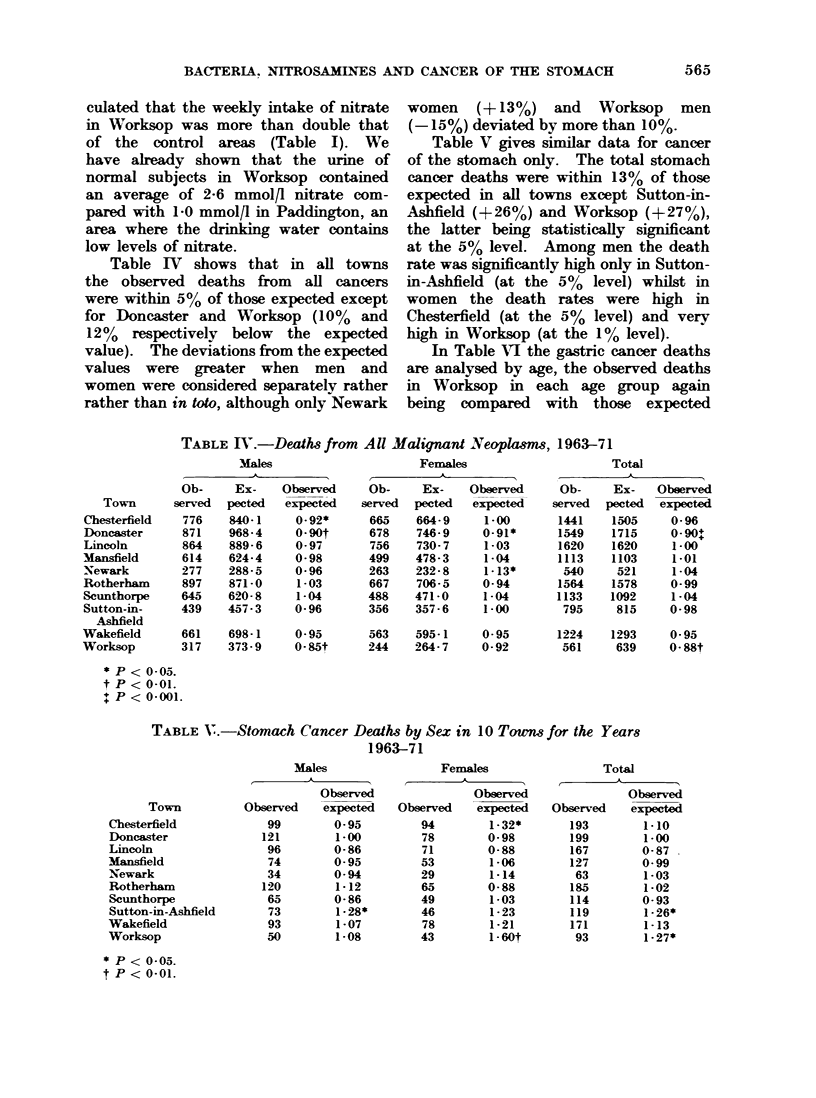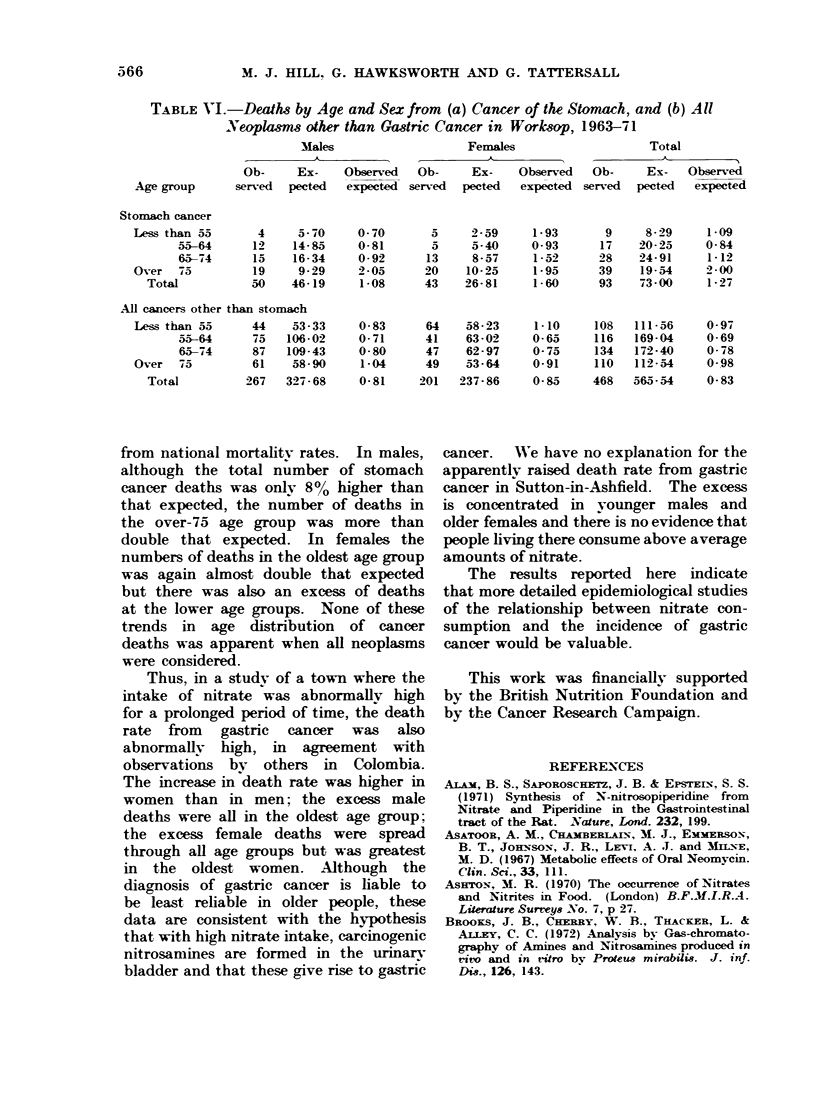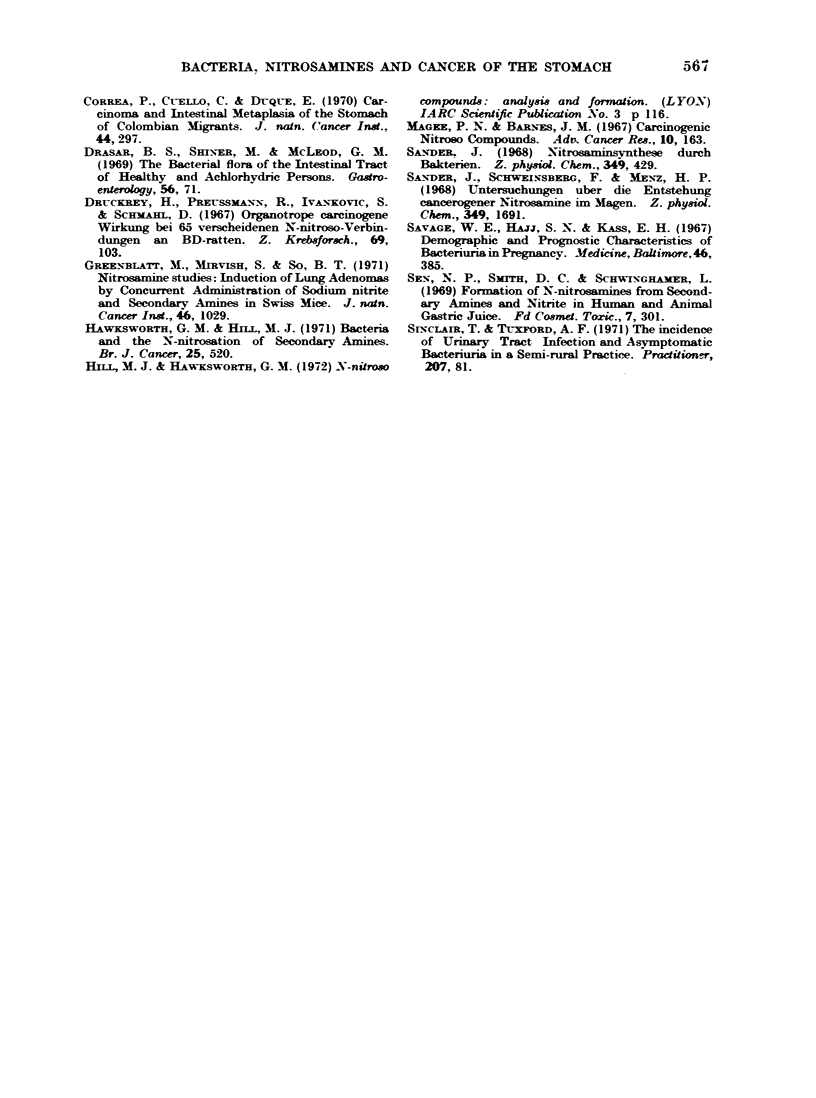# Bacteria, Nitrosamines and Cancer of the Stomach

**DOI:** 10.1038/bjc.1973.186

**Published:** 1973-12

**Authors:** M. J. Hill, G. Hawksworth, G. Tattersall

## Abstract

Until recently the public water supply to Worksop contained high concentrations of nitrate. An epidemiological study has revealed that, compared with low nitrate control towns, Worksop has an increased death rate from gastric cancer. The possible role of the bacterial production of nitrosamines in the aetiology of these stomach cancer deaths is discussed.


Br. J. Cancer (1973) **28**, 562

### BACTERIA, NITROSAMINES AND CANCER OF THE STOMACH

M. J. HILL, G. HAWKSWORTH AND G. TATTERSALL*

*From the Department of Bacteriology, St Mary's Hospital Medical School, London W2 1PG and the Medical Officer of Health, Worksop**

Received 15 August 1972 Accepted 18 August 1973

**Summary.—Until recently the public water supply to Worksop contained high concentrations of nitrate. An epidemiological study has revealed that, compared with low nitrate control towns, Worksop has an increased death rate from gastric cancer. The possible role of the bacterial production of nitrosamines in the aetiology of these stomach cancer deaths is discussed.**

N-NITROSAMINES are a potent group of carcinogens when administered to laboratory animals (Magee and Barnes, 1967; Druckrey *et al.*, 1967). For them to be of significance in human cancer they must be either (a) present in the environment and ingested with food, inhaled with vapours, etc. or (b) formed *in situ* in the human body from the parent amine.

To date, most of the work on the *in vivo* formation of nitrosamines has been concentrated on the possibility of an acid catalysed reaction taking place in the stomach between the parent amine and nitrite (Sen, Smith and Schwinghamer, 1967; Sander, Schweinsberg and Menz, 1968; Greenblatt, Mirvish and So, 1971). However, the reaction can also take place at physiological pH values catalysed by bacteria (Sander, 1968; Hawksworth and Hill, 1971) and consequently nitrosamines may be produced at any site where bacteria, secondary amine and nitrate or nitrite are present together. Our studies, which indicate rapid absorption of nitrate from the small intestine and subsequent excretion in the urine (Hawksworth and Hill, 1971), suggest that these conditions are unlikely to occur in the large bowel but might occur in individuals with bladder infections who happen to ingest large amounts of nitrate, and also in individuals with gastric achlorhydria.

People with gastric achlorhydria or anacidity have a profuse gastric flora (Drasar, Shiner and McCleod), 1969; these bacteria could then produce nitrosamines from ingested secondary amine (present in fish etc) and nitrate or nitrite. This has been demonstrated *in vivo* in rats (Alam, Saporoschetz and Epstein, 1971) whose poor gastric acid production permits a flora similar to that in achlorhydric man. The amount of nitrosamine formed will, however, be very small since the numbers of bacteria in gastric juice do not normally exceed $10^6$–$10^7$ per ml, the amounts of secondary amines in the diet are very small and the incubation time will be short. Achlorhydria is common in both men and women over the age of 50, but good data on the actual prevalence are not readily available.

Dietary nitrate is excreted in the urine and the urinary concentration is dependent, inevitably, on the amount ingested (Hawksworth and Hill, 1971). Thus in Paddington, where the estimated nitrate intake is 400–450 mg per week, the urinary nitrate concentration was 1·0 mmol/l, whilst in Worksop, where the nitrate intake was about 1000 mg per week the urinary nitrate concentration was 2·6 mmol/l. Dietary secondary amine is also excreted in the urine but the amount is small compared with that of the secondary amine produced in the gut, absorbed and excreted in the urine (Asatoor *et al.*, 1967). Potentially much more nitrosamine can be produced in the infected urinary bladder than in the achlorhydric stomach because there are more bacteria (with numbers exceeding $10^9$ per ml in 75% of cases: Savage, Hajj and Kass, 1967), much higher concentrations of secondary amine and longer incubation times. The amount of nitrosamine formed will be limited by the urinary nitrate concentration, which must exceed that of the secondary amines for nitrosation to take place (Hawksworth and Hill, 1971). We have demonstrated that nitrosamines are formed *in vivo* in the bladders of rats with experimental bladder infection (Hill and Hawksworth, 1972) and Brooks *et al.* (1972) have demonstrated nitrosamines in the urine of 2 people with urinary tract infections. A survey showed that the incidence of urinary tract infection in a rural general practice in England was 184 per 1000 patients per annum (Sinclair and Tuxford, 1971); most of these infections are due to *Escherichia coli* (Savage *et al.*, 1967) which is also the best nitrosating species (Hawksworth and Hill, 1971). They are common in women of child-bearing age and the incidence increases with age (Savage *et al.*, 1967); they are also common in men over the age of 50 (in association with infected prostates).

Nitrosamines act on target organs, which are characteristic of the nitrosamine and of the test animal used (Magee and Barnes, 1967). If nitrosamines are formed as described above, those most likely to be formed are dimethylnitrosamine (in both the stomach and the bladder), N-nitrosopiperidine and N-nitrosopyrrolidine (both in the bladder). There are no data on the target organs of these or any other nitrosamines in man. The bacterial formation of nitrosamines would be expected to produce cancer in men only in the oldest age group (since colonization of the bladder and stomach is rare in men below the age of 50) whereas in women the lower age groups might be affected more often since bladder infections in young women are fairly common.

Estimates have been made of the daily nitrate intake (Ashton, 1970). In general, food from vegetables, processed meat etc. contributes 70–80% of the total 400–500 mg/week, the remainder being from drinking water (Table I). It is difficult to locate populations ingesting unusually large amounts of nitrate in their food, but in areas with nitrate levels in the drinking water around the maximum level considered acceptable by the World Health Organization (*i.e.* 100 parts/$10^6$ of nitrate) the total intake is increased to more than 1000 mg per week with the water contributing 70% of this total. Narino in Colombia has a drinking water supply containing high levels of nitrate and has a high incidence

TABLE I.—*Estimated Weekly Nitrate Consumption of People Living in Normal Control Towns and in Worksop*

| Source | Weekly intake* | Control towns: Nitrate (parts/$10^6$) | Control towns: Weekly nitrate intake (mg) | Worksop: Nitrate (parts/$10^6$) | Worksop: Weekly nitrate intake (mg) |
|---|---|---|---|---|---|
| Meat | 220 g | 500 | 110 | 500 | 110 |
| Vegetables (excluding potatoes) | 450 g | 500 | 225 | 500 | 225 |
| Water | 7 l | 15 | 105 | 93 | 645 |
| Total | | | 440 | | 980 |

* Ashton (1970).

of cancer of the stomach; the increase is greater in women then in men (Correa, Cuello and Duque, 1970) but there is no information on the relative increases at various ages. Until recently, and at least since 1953, the drinking water at Worksop contained an average of 90 mg/l nitrate, the highest level in any borough in the United Kingdom. Although the town now has a different source of water with a low nitrate content, it seemed suitable for a retrospective epidemiological study.

A preliminary analysis of deaths in Worksop from cancer in the years 1958–71 suggested that the death rates from cancer of the stomach and liver might be abnormally high in the town (Table II). The Office of Population, Censuses and Surveys (OPCS) supplies each year to the Medical Officers of Health in all local authority areas details of deaths occurring in each area, tabulated by age, sex and a restricted list of sites (W.H.O. abbreviated list), including cancer of the stomach but not cancer of the liver. The OPCS kindly made copies of these tabulations available to us for the years 1963–71 for Worksop and a number of control towns selected for their proximity to Worksop and their similar social class structure as determined from the 1966 census (Table III). "Expected" numbers of deaths in each town were calculated using national age and sex specific mortality rates for the corresponding time period. The nitrate content of the drinking water of the control towns was less than 10 mg/l compared with a mean value of 90 mg/l for the nitrate content of Worksop water for the relevant period. On the assumptions regarding food and water consumption reported by Ashton (1970), it was cal-

TABLE II.—*Cancer Deaths in Worksop* (1958–71) *Compared with Those Expected*

| | Males | | | Females | | |
|---|---|---|---|---|---|---|
| Site | Expected* | Observed† | Observed/Expected | Expected* | Observed† | Observed/Expected |
| Stomach | 70 | 92 | 1·31 | 43 | 83 | 1·93 |
| Oesophagus | 10·4 | 14 | 1·34 | 8·0 | 10 | 1·25 |
| Liver | 1·8 | 10 | 5·56 | 1·4 | 8 | 5·72 |
| Bladder | 39 | 37 | 0·95 | 12 | 12 | 1·00 |
| Breast | | | | 133 | 119 | 0·90 |

* Expected number of deaths calculated from the age adjusted rates for the Sheffield registry and from the age distribution of the population.

† Observed numbers of deaths taken from the records at the Public Health Department, Worksop.

TABLE III.—*Socioeconomic Classification of the Populations of the Towns Studied. Data Obtained from the* 1966 *Sample Census*

| Town | Employers, managers and professional workers | Skilled workers | Non-manual workers | Semi-skilled and agricultural workers | Unskilled manual workers | Others |
|---|---|---|---|---|---|---|
| Chesterfield | 11·0 | 45·2 | 14·7 | 18·1 | 10·5 | 0·7 |
| Doncaster | 12·0 | 39·6 | 16·6 | 20·5 | 8·5 | 2·8 |
| Lincoln | 10·2 | 43·6 | 19·3 | 17·3 | 7·1 | 2·6 |
| Mansfield | 13·0 | 41·1 | 12·2 | 26·2 | 6·7 | 0·8 |
| Newark | 11·0 | 42·1 | 17·5 | 16·9 | 10·3 | 2·3 |
| Rotherham | 8·9 | 47·4 | 12·0 | 17·6 | 12·7 | 1·5 |
| Scunthorpe | 8·4 | 50·2 | 13·0 | 15·1 | 12·1 | 1·3 |
| Sutton-in-Ashfield | 7·0 | 47·2 | 8·6 | 29·2 | 6·6 | 1·4 |
| Wakefield | 14·6 | 39·2 | 15·9 | 20·8 | 8·8 | — |
| Worksop | 9·6 | 42·3 | 12·3 | 26·1 | 8·3 | 1·4 |

culated that the weekly intake of nitrate in Worksop was more than double that of the control areas (Table I). We have already shown that the urine of normal subjects in Worksop contained an average of 2·6 mmol/l nitrate compared with 1·0 mmol/l in Paddington, an area where the drinking water contains low levels of nitrate.

Table IV shows that in all towns the observed deaths from all cancers were within 5% of those expected except for Doncaster and Worksop (10% and 12% respectively below the expected value). The deviations from the expected values were greater when men and women were considered separately rather rather than *in toto*, although only Newark women (+13%) and Worksop men (−15%) deviated by more than 10%.

Table V gives similar data for cancer of the stomach only. The total stomach cancer deaths were within 13% of those expected in all towns except Sutton-in-Ashfield (+26%) and Worksop (+27%), the latter being statistically significant at the 5% level. Among men the death rate was significantly high only in Sutton-in-Ashfield (at the 5% level) whilst in women the death rates were high in Chesterfield (at the 5% level) and very high in Worksop (at the 1% level).

In Table VI the gastric cancer deaths are analysed by age, the observed deaths in Worksop in each age group again being compared with those expected

TABLE IV.—*Deaths from All Malignant Neoplasms*, 1963–71

| | Males | | | Females | | | Total | | |
|---|---|---|---|---|---|---|---|---|---|
| Town | Observed | Expected | Observed/expected | Observed | Expected | Observed/expected | Observed | Expected | Observed/expected |
| Chesterfield | 776 | 840·1 | 0·92* | 665 | 664·9 | 1·00 | 1441 | 1505 | 0·96 |
| Doncaster | 871 | 968·4 | 0·90† | 678 | 746·9 | 0·91* | 1549 | 1715 | 0·90‡ |
| Lincoln | 864 | 889·6 | 0·97 | 756 | 730·7 | 1·03 | 1620 | 1620 | 1·00 |
| Mansfield | 614 | 624·4 | 0·98 | 499 | 478·3 | 1·04 | 1113 | 1103 | 1·01 |
| Newark | 277 | 288·5 | 0·96 | 263 | 232·8 | 1·13* | 540 | 521 | 1·04 |
| Rotherham | 897 | 871·0 | 1·03 | 667 | 706·5 | 0·94 | 1564 | 1578 | 0·99 |
| Scunthorpe | 645 | 620·8 | 1·04 | 488 | 471·0 | 1·04 | 1133 | 1092 | 1·04 |
| Sutton-in-Ashfield | 439 | 457·3 | 0·96 | 356 | 357·6 | 1·00 | 795 | 815 | 0·98 |
| Wakefield | 661 | 698·1 | 0·95 | 563 | 595·1 | 0·95 | 1224 | 1293 | 0·95 |
| Worksop | 317 | 373·9 | 0·85† | 244 | 264·7 | 0·92 | 561 | 639 | 0·88† |

* $P < 0·05$.

† $P < 0·01$.

‡ $P < 0·001$.

TABLE V.—*Stomach Cancer Deaths by Sex in* 10 *Towns for the Years* 1963–71

| | Males | | Females | | Total | |
|---|---|---|---|---|---|---|
| Town | Observed | Observed/expected | Observed | Observed/expected | Observed | Observed/expected |
| Chesterfield | 99 | 0·95 | 94 | 1·32* | 193 | 1·10 |
| Doncaster | 121 | 1·00 | 78 | 0·98 | 199 | 1·00 |
| Lincoln | 96 | 0·86 | 71 | 0·88 | 167 | 0·87 |
| Mansfield | 74 | 0·95 | 53 | 1·06 | 127 | 0·99 |
| Newark | 34 | 0·94 | 29 | 1·14 | 63 | 1·03 |
| Rotherham | 120 | 1·12 | 65 | 0·88 | 185 | 1·02 |
| Scunthorpe | 65 | 0·86 | 49 | 1·03 | 114 | 0·93 |
| Sutton-in-Ashfield | 73 | 1·28* | 46 | 1·23 | 119 | 1·26* |
| Wakefield | 93 | 1·07 | 78 | 1·21 | 171 | 1·13 |
| Worksop | 50 | 1·08 | 43 | 1·60† | 93 | 1·27* |

* $P < 0·05$.
† $P < 0·01$.

TABLE VI.—*Deaths by Age and Sex from (a) Cancer of the Stomach, and (b) All Neoplasms other than Gastric Cancer in Worksop*, 1963–71

| | Males | | | Females | | | Total | | |
|---|---|---|---|---|---|---|---|---|---|
| Age group | Observed | Expected | Observed / expected | Observed | Expected | Observed / expected | Observed | Expected | Observed / expected |
| Stomach cancer | | | | | | | | | |
| Less than 55 | 4 | 5·70 | 0·70 | 5 | 2·59 | 1·93 | 9 | 8·29 | 1·09 |
| 55–64 | 12 | 14·85 | 0·81 | 5 | 5·40 | 0·93 | 17 | 20·25 | 0·84 |
| 65–74 | 15 | 16·34 | 0·92 | 13 | 8·57 | 1·52 | 28 | 24·91 | 1·12 |
| Over 75 | 19 | 9·29 | 2·05 | 20 | 10·25 | 1·95 | 39 | 19·54 | 2·00 |
| Total | 50 | 46·19 | 1·08 | 43 | 26·81 | 1·60 | 93 | 73·00 | 1·27 |
| All cancers other than stomach | | | | | | | | | |
| Less than 55 | 44 | 53·33 | 0·83 | 64 | 58·23 | 1·10 | 108 | 111·56 | 0·97 |
| 55–64 | 75 | 106·02 | 0·71 | 41 | 63·02 | 0·65 | 116 | 169·04 | 0·69 |
| 65–74 | 87 | 109·43 | 0·80 | 47 | 62·97 | 0·75 | 134 | 172·40 | 0·78 |
| Over 75 | 61 | 58·90 | 1·04 | 49 | 53·64 | 0·91 | 110 | 112·54 | 0·98 |
| Total | 267 | 327·68 | 0·81 | 201 | 237·86 | 0·85 | 468 | 565·54 | 0·83 |

from national mortality rates. In males, although the total number of stomach cancer deaths was only 8% higher than that expected, the number of deaths in the over-75 age group was more than double that expected. In females the numbers of deaths in the oldest age group was again almost double that expected but there was also an excess of deaths at the lower age groups. None of these trends in age distribution of cancer deaths was apparent when all neoplasms were considered.

Thus, in a study of a town where the intake of nitrate was abnormally high for a prolonged period of time, the death rate from gastric cancer was also abnormally high, in agreement with observations by others in Colombia. The increase in death rate was higher in women than in men; the excess male deaths were all in the oldest age group; the excess female deaths were spread through all age groups but was greatest in the oldest women. Although the diagnosis of gastric cancer is liable to be least reliable in older people, these data are consistent with the hypothesis that with high nitrate intake, carcinogenic nitrosamines are formed in the urinary bladder and that these give rise to gastric cancer. We have no explanation for the apparently raised death rate from gastric cancer in Sutton-in-Ashfield. The excess is concentrated in younger males and older females and there is no evidence that people living there consume above average amounts of nitrate.

The results reported here indicate that more detailed epidemiological studies of the relationship between nitrate consumption and the incidence of gastric cancer would be valuable.

This work was financially supported by the British Nutrition Foundation and by the Cancer Research Campaign.

### REFERENCES

ALAM, B. S., SAPOROSCHETZ, J. B. & EPSTEIN, S. S. (1971) Synthesis of N-nitrosopiperidine from Nitrate and Piperidine in the Gastrointestinal tract of the Rat. *Nature, Lond.* **232**, 199.

ASATOOR, A. M., CHAMBERLAIN, M. J., EMMERSON, B. T., JOHNSON, J. R., LEVI, A. J. and MILNE, M. D. (1967) Metabolic effects of Oral Neomycin. *Clin. Sci.*, **33**, 111.

ASHTON, M. R. (1970) The occurrence of Nitrates and Nitrites in Food. (London) *B.F.M.I.R.A. Literature Surveys No.* 7, p 27.

BROOKS, J. B., CHERRY, W. B., THACKER, L. & ALLEY, C. C. (1972) Analysis by Gas-chromatography of Amines and Nitrosamines produced *in vivo* and *in vitro* by *Proteus mirabilis*. *J. inf. Dis.*, **126**, 143.

CORREA, P., CUELLO, C. & DUQUE, E. (1970) Carcinoma and Intestinal Metaplasia of the Stomach of Colombian Migrants. *J. natn. Cancer Inst.*, **44**, 297.
DRASAR, B. S., SHINER, M. & McLEOD, G. M. (1969) The Bacterial flora of the Intestinal Tract of Healthy and Achlorhydric Persons. *Gastroenterology*, **56**, 71.
DRUCKREY, H., PREUSSMANN, R., IVANKOVIC, S. & SCHMAHL, D. (1967) Organotrope carcinogene Wirkung bei 65 verscheidenen N-nitroso-Verbindungen an BD-ratten. *Z. Krebsforsch.*, **69**, 103.
GREENBLATT, M., MIRVISH, S. & So, B. T. (1971) Nitrosamine studies: Induction of Lung Adenomas by Concurrent Administration of Sodium nitrite and Secondary Amines in Swiss Mice. *J. natn. Cancer Inst.*, **46**, 1029.
HAWKSWORTH, G. M. & HILL, M. J. (1971) Bacteria and the N-nitrosation of Secondary Amines. *Br. J. Cancer*, **25**, 520.
HILL, M. J. & HAWKSWORTH, G. M. (1972) *N-nitroso compounds: analysis and formation.* (*LYON*) *IARC Scientific Publication No.* 3 p 116.
MAGEE, P. N. & BARNES, J. M. (1967) Carcinogenic Nitroso Compounds. *Adv. Cancer Res.*, **10**, 163.
SANDER, J. (1968) Nitrosaminsynthese durch Bakterien. *Z. physiol. Chem.*, **349**, 429.
SANDER, J., SCHWEINSBERG, F. & MENZ, H. P. (1968) Untersuchungen uber die Entstehung cancerogener Nitrosamine im Magen. *Z. physiol. Chem.*, **349**, 1691.
SAVAGE, W. E., HAJJ, S. N. & KASS, E. H. (1967) Demographic and Prognostic Characteristics of Bacteriuria in Pregnancy. *Medicine, Baltimore*, **46**, 385.
SEN, N. P., SMITH, D. C. & SCHWINGHAMER, L. (1969) Formation of N-nitrosamines from Secondary Amines and Nitrite in Human and Animal Gastric Juice. *Fd Cosmet. Toxic.*, **7**, 301.
SINCLAIR, T. & TUXFORD, A. F. (1971) The incidence of Urinary Tract Infection and Asymptomatic Bacteriuria in a Semi-rural Practice. *Practitioner*, **207**, 81.